# Evaluation of alveolar bone loss among diabetic patients with class II amalgam restorations

**DOI:** 10.6026/973206300200798

**Published:** 2024-07-31

**Authors:** Abdo Mohammed Mohammed Abdulrazzaq, Sultan Alanazi

**Affiliations:** 1 Department of Preventive Dental Science, Faculty of Dentistry, Najran University, Najran, Saudi Arabia

**Keywords:** Dental class II amalgam restorations, alveolar bone loss, plaque index, gingival Index, diabetic, non-diabetic

## Abstract

The relationship between Class II amalgam restorations and alveolar bone loss among diabetic and non-diabetic patients is evaluated at the Faculty of Dentistry, Najran University, KSA. Hence, we compared type 2 diabetic (n = 32) and non-diabetic patients (n=32) using clinical assessments and imaging techniques. Parameters such as bone loss rate, extent, age, and periodontal condition (plaque index and Gingival Index) were analyzed. Analysis of data shows that diabetic patients on average have higher bone loss.

## Background:

In oral health, dental restorations serve as key instruments in the preservation and restoration of the oral cavity's function and aesthetics [1]. A key concern in dental interventions is how restorations affect alveolar bone health, this inquiry takes on particular significance when considering patients with diabetes, a chronic metabolic disorder marked by elevated blood glucose levels, which holds the potential to affect an array of physiological processes, including those that pertain to oral health [2]. The alveolar bone supports and sustains teeth, it is crucial for dental stability and function, however alveolar bone loss, often intertwined with conditions such as periodontal disease, has shown an amplified prevalence in individuals grappling with diabetes, manifesting a nuanced interplay between systemic health and oral well-being [3]. The effect of dental restorations on alveolar bone loss is complex, intriguing and it is a key subject in periodontal health, dental restorations, such as fillings, crowns, implants, dentures, bridges and orthodontic appliances, have consistently demonstrated the ability to preserve alveolar bone by mimicking natural tooth structures and providing functional loading that stimulates the surrounding bone [4]. Researchers investigated bone loss and implant stability in patients with and without hyperglycemia, the study demonstrated that T2DM patients exhibited significantly higher PIBL, particularly around exposed single-tooth dental implant supported restorations, suggesting the influence of immune-inflammatory factors in diabetes-related PIBL [5]. Researchers did a multi-center study on direct restorations, they looked at factors affecting their survival, the study revealed differences in the failure rates based on restoration class, patient age, check-up frequency, and restorative material, these findings provide practical insights for dental practitioners to improve the longevity of direct restorations and tailor treatments to individual patient needs [6]. Writers reviewed causes of marginal bone changes around implants, their findings challenge the idea that bone loss always means disease, and they suggest it can be a natural part of healing and adaptation, this nuanced understanding can help avoid unnecessary concerns about implant health in cases where marginal bone loss is within certain limits [7]. The service life of class II amalgam restorations and participant age consistently correlated with worse periodontal parameters, indicating these as the most influential risk factors [8]. Therefore, it is of interest to establish the relationship between class II amalgam dental restorations and the loss of alveolar bone among diabetic and non-diabetic patients.

## Materials and Methods:

The researchers obtained ethical approval from the University's Research Ethics Committee, following all requirements. Clinical examinations and evaluations involving humans adhered to the ethical standards of the institutional/national research committee and the Helsinki Declaration (amended by the 64th WMA General Assembly, Fortaleza, Brazil, 2013) and its subsequent amendments or comparable standards.

## Group allocation participants:

In this observational comparative cross-sectional study, we selected 64 type 2 diabetes and non- diabetic patients from a sample of 489 patients at the Faculty of Dentistry, Najran University, Saudi Arabia, were divided into two groups. Each group comprised 32 patients, aged 45-60 years. Group 1 (G1) diabetic patients had class II amalgam restorations, Group 2 (G2) non- diabetic patients (control group), had class II amalgam restorations. The service life of all restorations ranged from 7 to 10 years. Clinical examinations included plaque index (PI) and gingival index (GI) assessments, radiographic evaluation of overhang restorations, along with alveolar bone loss measurements, using panoramic X-ray, were carried out for each group by 2 clinicians. We used Crano and Brewer's formula [9] for medical research sample size calculation n = Nn*/ N + n*, the initial estimated sample size (n*) was calculated using the formula n* = P (1 - P)/ (SE) 2, P was assumed to be 0.5 to maximize the sample size, representing the estimated proportion of participants and SE, representing the standard error, was assumed to be 0.05.

## Inclusion:

## The inclusion criteria in the selection of the first group of diabetic patients were included:

[1] Patients with type 2 diabetes for 2 years or more

[2] HbA1c levels ≥ 7%;

[3] 45 - 60 years of age

[4] A minimum of 12 teeth remaining.

[5] The class II amalgam dental restorations life range 7 - 10 years.

## The inclusion criteria in the selection of the second group of non-diabetic patients were included:

[1] Medically fit

[2] 45 - 60 years of age

[3] A minimum of12 teeth remaining.

[4] The class II amalgam dental restorations' life ranges 7 - 10 years.7 - 10 years.

## Exclusion:

[1] Infectious diseases, HIV, acquired and (or) congenital heart diseases, Hypertension hepatic disease, kidney diseases, or epilepsy.

[2] Use of antibiotic or steroid therapy for previous two week before retrieving the data

[3] Receiving immunomodulatory drugs

[4] Receiving periodontal treatment for the previous six months

[5] Elderly patients without teeth

[6] Dental crowding or occlusal trauma.

## Clinical examinations:

## Teeth examination:

In the dental examination, clinicians used a charting system to systematically document carious lesions (C), missing teeth (X), mobile teeth (M), fractures (#), restorations (R), crowns (Cr), and bridge-anchoring teeth (Br). This systematic approach enhanced the study's credibility and utility in dental research and patient care while ensuring ethical compliance.

## Periodontal parameters examination:

Two clinicians were evaluated plaque accumulation and the state of gingival inflammation in each participant. Each participant received a comprehensive oral clinical examination to assess the condition of their periodontal tissues. The clinical evaluation relied on the participants' present dental condition, and no dental procedures were preceding this examination. The clinical screening examination included evaluating periodontal tissue condition by assessing dental plaque using PI and the qualitative state of the gingiva using GI. The assessments used scoring systems proposed for Plaque Index [10^10^] and Gingival Index [11]. Every tooth surface (labial or facial, lingual, or palatal, mesial, and distal,) inspected for each participant, except for third molars, using a William's periodontal probe.

## Radiographic evaluation:

Clinicians conducted Orthopantomography or Panorex (OPG) X-ray assessment to examine the extent of alveolar bone loss and identify any signs of irregular or overhanging restorations, which may indicate potential problems with the dental restorations. They employed a panoramic Xray unit, adjusting the resolution and height according to patient requirements. The patient's chin was secured, and the occlusal plane was set horizontally, with adjustments made using a laser and touch screen. Final volume location adjustments were made as needed. A computer-assisted system used for digitizing and analyzing radiographs for linear measurements. These assessments were carried out during routine dental practice visits.

## Alveolar bone loss measurements:

Panoramic X-ray imaging was employed to measure alveolar bone loss. This was a critical aspect of the study to determine the impact of different restorations on bone health in diabetic and non-diabetic patients.

The acceptability of OPG radiographs determined based on specific criteria, which were as follows:

[1] Adequate visibility of anatomical features such as the Cementoenamel Junction (CEJ), Alveolar Bone Crest (ABC), and tooth Apices (AP).

[2] The CEJs should not be compromised by factors like restorations, prostheses, overlapping images, or image defects.

[3] Both proximal areas (mesial and distal) must be measurable.

## The formula of calculate the percentage of alveolar bone loss:

The authors used the following formula to calculate the percentage of bone loss around a tooth: ((CEJ-ABC) - 2mm) / ((CEJ-AP) - 2mm) x 100. The formula appears to be based on measurements of two reference points: CEJ (Cementoenamel Junction) to ABC (Alveolar Bone Crest) and CEJ to AP (Alveolar Process).

## Results:

64 persons were enrolled in this study, out of which 32 (50%) were diabetic patients had class II amalgam restorations (test) Group 1 (G1) , and 32 (50%) non- diabetic patients(control group)Group 2 (G2) to compare diabetic (G1) and non-diabetic (G2) patients treated with Class II amalgam restorations, data were statistically analyzed using SPSS 18 software. Two tests were employed: the Two-Sample T-Test and Descriptive Statistics. The results for both groups were then interpreted. The variations were statistically significant (Table 1 and Table 2).

## Analysis:

Regarding age, the T-Value was 1.29, and the P-Value was 0.201, indicating no statistically significant difference in average age between non-diabetic and diabetic patients. For the Plaque Index (P.I.), the T-Value was -4.61, and the P-Value was < 0.001, showing a statistically significant difference with diabetic patients having a higher average P.I. The Gingival Index (G.I.) had a T-Value of -4.47 and a P-Value of < 0.001, indicating a statistically significant difference, with diabetic patients having a higher average G.I. The Life of Restoration showed a T-Value of -2.17 and a P-Value of 0.033, indicating a statistically significant difference, with diabetic patients having restorations for a slightly longer average duration. Bone Loss (%) had a T-Value of -4.83 and a P-Value of < 0.001, indicating a statistically significant difference, with diabetic patients experiencing greater average bone loss.

## Interpretation:

Diabetic patients show higher average bone loss (53.656%) compared to non-diabetic patients (49.25%). This increased bone loss may result from higher periodontal indices (P.I. 2.72, G.I. 2.52) and systemic health issues like elevated HbA1c levels, which affect bone density and healing. The average lifespan of restorations is similar for both groups (8.23 years for non-diabetics, 8.85 years for diabetics), suggesting factors other than restoration duration influence bone loss. Poorer periodontal health in diabetics, with higher P.I. and G.I. scores, correlates with increased bone loss. Therefore, higher bone loss in diabetics is likely due to diabetes impacting healing and bone density maintenance, rather than the age of restorations.

## Discussion:

The observed outcomes, in conjunction with the present findings, may suggest a correlation between periodontal scores in diabetic patients and the duration of hyperglycemia. However, it's crucial to note that this influence could vary significantly due to the inclusion of other potential risk factors, including poor oral hygiene, defective dental restorations, and advanced age. During clinical examination, the majority of patients in both diabetic and non-diabetic groups exhibited poor oral hygiene. Additionally, the mean Plaque Index (PI) and Gingival Index (GI) scores were notably high in both groups under investigation. The elevated oral health scores, indicating poorer periodontal parameters, coupled with the observed poor oral health status, underscore the prevalence of severe chronic periodontitis among subjects in both groups. Consequently, it must be acknowledged as a substantial predisposing factor contributing to their inferior periodontal parameters. In the current study, patients with diabetes who underwent treatment with Class II amalgam restorations exhibited mean values of alveolar bone loss that surpassed 50%. In this study, patients in group G2 exhibited the most adverse periodontal parameters, attributable to multiple risk factors: 1) Excessive forces from Class II amalgam restorations; 2) Diabetes mellitus; 3) Poor oral hygiene; 4) Duration of Class II amalgam restorations; and 5) Patient age. It was anticipated that this group would demonstrate significantly higher values due to the accumulation of more risk factors associated with this treatment compared to G1 non-diabetic patients. An overhanging restoration, when present, can synergistically interact with other factors, leading to pronounced alveolar bone loss, as evidenced in this study. Previous literature has demonstrated a wide variation in the prevalence of Class II amalgam restorations, ranging from (16.5% - 76%) [12]. Regarding chronic diseases or systemic factors, the current study lacks precise data on the onset of diabetes and the methods used to manage diabetes (treatment). Additionally, we did not evaluate the obesity status of the subjects. Obesity is widely recognized as a risk factor for periodontal disease and may contribute to poorer periodontal parameters [13]. Certain limitations associated with dental restorations merit consideration, including the smoothness of Class II restorations and the dimensions of proximal overhangs.

## Conclusion:

Data shows significant differences between diabetic and non-diabetic patients, highlighting diabetes' substantial impact on periodontal health and bone loss in those with Class II amalgam restorations. Diabetics (G1) showed severe periodontal damage with over 50% alveolar bone loss and higher PI and GI scores, likely due to chronic periodontitis. Non-diabetic patients (G2) had better periodontal health with less than 50% bone loss and lower PI and GI scores. Managing periodontal health in diabetic patients is crucial due to their heightened risk of bone loss, necessitating regular dental check-ups, rigorous oral hygiene, and potential adjunctive therapies.

## Figures and Tables

**Table 1:** Descriptive Statistics

**Group**	**No. of Patients**	**Age (Mean ± SD)**	**P.I (Mean ± SD)**	**G.I (Mean ± SD)**	**Life of Restoration (Mean ± SD)**	**Bone Loss (%) (Mean ± SD)**
Non-Diabetic (G2)	32	52 ± 4.31	2.58 ± 0.11	2.38 ± 0.12	8.23 ± 1.12	49.25 ± 3.12
Diabetic (G1)	32	50.5 ± 4.15	2.72 ± 0.10	2.52 ± 0.11	8.85 ± 1.05	53.656 ± 3.45

**Table 2:** Two-Sample T-Test Results

**Variable**	**T-Value**	**P-Value**	**Significant difference?**
Age	1.29	0.201	No
Plaque Index (P.I)	-4.61	< 0.001	Yes
Gingival Index (G.I)	-4.47	< 0.001	Yes
Life of Restoration (years)	-2.17	0.033	Yes
Bone Loss (%)	-4.83	< 0.001	Yes
